# Imaging Findings of Gastric Diverticula

**DOI:** 10.1155/2014/923098

**Published:** 2014-10-23

**Authors:** Dominik Schramm, Andreas Gunter Bach, Alexander Zipprich, Alexey Surov

**Affiliations:** ^1^Department of Radiology, Martin Luther University of Halle-Wittenberg, Ernst-Grube Straße 40, 06097 Halle, Germany; ^2^Department of Gastroenterology, Martin Luther University of Halle-Wittenberg, Ernst-Grube Straße 40, 06097 Halle, Germany

## Abstract

*Introduction*. Gastric diverticula (GD) are very rare. Computer tomographic findings in GD have been reported only as case reports previously. The aim of this study was to estimate the prevalence of GD on computed tomography (CT) and to analyze their radiological appearances. *Materials and Methods*. From 2006 to 2013, a total of 14,428 patients were examined by abdominal/thoracic CT at our institution. GD were diagnosed in 18 (0.12%) patients (13 women and 5 men, median age, 64 years). In 9 patients, additional endoscopy and in 7 patients upper gastrointestinal investigation with contrast medium were performed. Magnetic resonance imaging (MRI) was available for 3 cases. *Results*. In all patients GD were diagnosed incidentally during CT examination. The diverticula were located at the posterior wall of the gastric fundus below the esophagogastric junction. On CT, GD presented as cystic lesions with a thin wall and an air fluid level, located behind the stomach between spleen, adrenal gland, and crus of the left diaphragm. *Conclusion*. The prevalence of GD encountered in our CT series is 0.12%. GD demonstrate typical CT appearances, namely, cystic lesions located in the left paravertebral region. The radiologist should be familiar with this finding to avoid possible misinterpretations.

## 1. Introduction

Gastric diverticula (GD) are very rare. According to previous reports, their prevalence ranges from 0.02% to 2.6% depending on applied diagnostic method [1–3]. There are two different types of GD: congenital and acquired [1, 4]. Congenital gastric diverticula (CGD) are true diverticula; that is, they contain all layers of the stomach wall [3, 4]. They are located typically in the cardia on the posterior gastric wall below the esophagogastral junction [2–4].

Acquired gastric diverticula (AGD) are false or pseudodiverticula, that is, pulsion-type herniation through the gaps in the muscular layer of the gastric wall, and are located usually in the antrum or in the pars pylorica of the stomach [2, 4]. According to the literature, most GD (approximately 70%) are CGD [1–3].

The endoscopic appearances or findings in upper gastrointestinal contrast investigation of GD are well documented in the literature [1, 3, 4].

Nowadays, the use of computed tomography (CT) or magnetic resonance imaging (MRI) for a variety of diagnostic pathways increases significantly. However, CT and/or MR findings in stomach diverticula have been reported only as case reports previously [5–7]. Furthermore, there are no data regarding the frequency of GD on CT or MRI.

Therefore, the aim of this study was to estimate the prevalence of GD in a large series of computed tomography investigations and to analyze their radiological appearances.

## 2. Materials and Methods

From January 2006 to December 2013 a total of 14,428 patients were examined by abdominal or thoracic and abdominal computed tomographic examinations at our institution. Computed tomography (Somatom Sensation 64, Siemens, Erlangen, Germany) was performed in all patients. In all cases 60–140 mL of iodinated intravenous contrast medium was given at a rate of 1.5–4.0 mL/s by a power injector (Medtron GmbH, Germany), with a scan delay of 30–90 s after onset of injection. When the abdominal and pelvic regions were investigated by CT, oral contrast material (1-2 L of a flavoured 3% diatrizoate meglumine solution, Bayer Vital, Berlin) was given 60–120 min before the examination. Typical imaging parameters were 120 kVp, 150–300 mAs, and 0.6 to 6 mm of slice thickness with a pitch of 0.8–1.2.

All CT images were reevaluated retrospectively by two radiologists (A.S. and D.S. with 11 and 4 years of general radiological experience, resp.). All images were analyzed in digital form using a PACS workstation (Centricity PACS, GE Medical Systems, Milwaukee, WI, USA).

In this time period, GD were diagnosed in 18 patients on CT. There were 13 women and 5 men with a median age of 64 years (range, 42–86 years; mean age, 65.5 ± 14.1 years).

Additional MRI was available for 3 patients with GD. MR imaging was performed using a 1.5T MRI scanner Magnetom Vision Sonata Upgrade, Siemens, Germany. MRI sequences included fat-suppressed T2W short tau inversion recovery (STIR) images, half-Fourier acquisition single-shot turbo spin echo (HASTE) images, T1 weighted (T1W) spin echo (T1W SE) images, and T1w flash 2D (FL 2D) images.

In 7 patients with GD upper gastrointestinal contrast examinations were performed after oral application of 30–70 mL of contrast medium (diatrizoate meglumine solution, Bayer Vital, Berlin).

In 9 patients gastroduodenoscopy was performed additionally.

All images of every patient were reanalysed for the present study.

For statistical analysis the SPSS statistical software package was used. Collected data were evaluated by means of descriptive statistics (absolute and relative frequencies). Continuous variables were expressed as mean ± standard deviation (SD) and categorical variables as percentages. Numbers of events between groups were compared with a chi-square test. Significance level was *P* < 0.05.

## 3. Results

The prevalence of GD in our study was 0.12% (18 of 14,428). In all patients GD were diagnosed incidentally during CT examination.

Most patients with GD were female; the female : male ratio was 2.6 : 1. The female patients were older (68.5 versus 57.8 years; *P* = 0.0682).

In all cases the identified diverticula were located at the posterior wall of the gastric fundus below the esophagogastric junction. The mean size of GD was 21.7 ± 10.9 mm (median size, 20 mm; range, 7–45 mm) in left-right (transversal) and 31.7 ± 18.9 mm (median size, 26 mm; range, 10–75 mm) in anterior-posterior (sagittal) direction.

The GD in transversal direction were larger in male patients, although statistically not significant (41.6 mm versus 27.8 mm; *P* = 0.1999).

**Figure 1:**
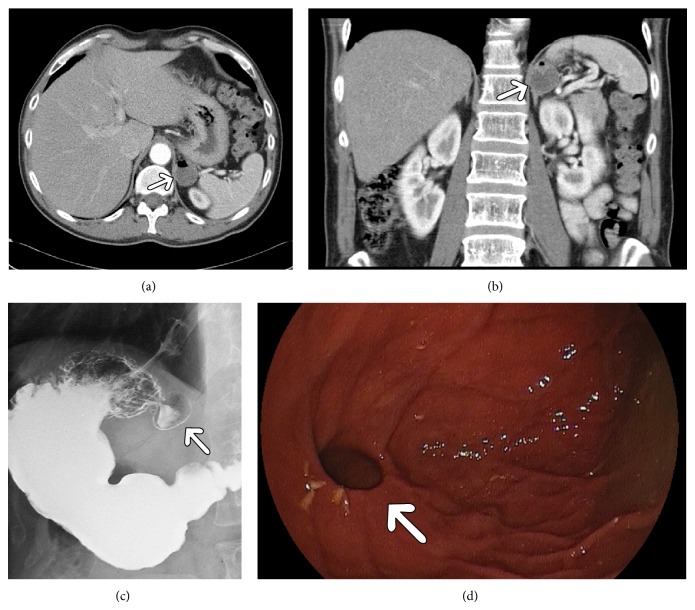
Imaging findings in a 57-year-old patient. (a) Computer tomographic scan detecting a mass in the left paravertebral region behind the stomach with air fluid level (arrow). (b) The lesion (arrow) in coronal CT reconstruction. (c) Upper gastrointestinal investigation with contrast showing a gastric diverticulum in the cardia on the posterior gastric wall below the esophagogastral junction (arrow). (d) Endoscopy finding with entry of the diverticulum (arrow).

**Figure 2:**
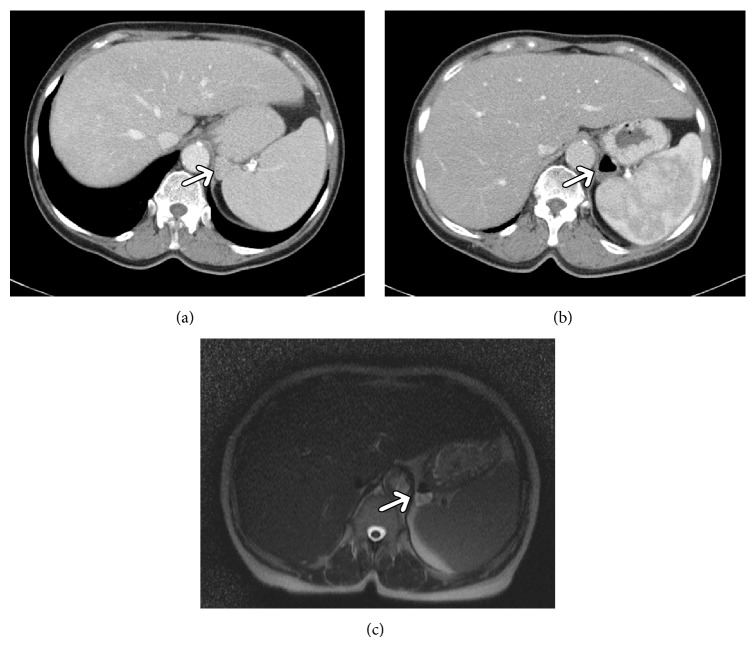
Imaging findings in a 70-year-old woman with known history of hepatocellular carcinoma. (a) Primary computer tomographic scan detecting a solid mass between the stomach aorta and spleen (arrow). The lesion was interpreted as an accessory spleen. (b) On computer tomographic scan 5 months after the primary investigation the lesion is cystic with air fluid level (arrow). (c) MRI image of the lesion (arrow).

**Figure 3:**
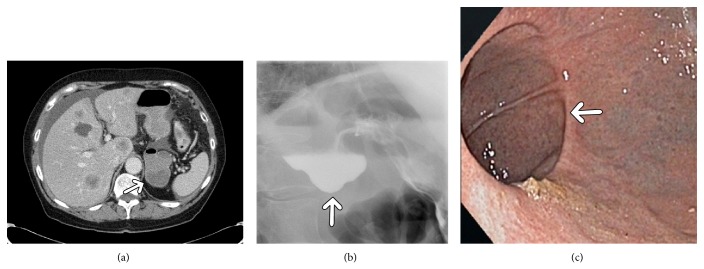
Imaging findings in a 60-year-old woman with known history of lung cancer. (a) Computer tomographic scans presenting a large gastric diverticulum (arrows). (b) Upper gastrointestinal investigation with contrast showing a gastric diverticulum in the cardia on the posterior gastric wall below the esophagogastral junction (arrow). (c) Endoscopy finding with entry of the diverticulum (arrow).

On CT, the diverticula presented as cystic lesions with thin wall and air fluid level located behind the stomach between the spleen, adrenal gland, and crus of the left diaphragm (Figures 1–3). In one case, the diverticulum showed no air in the first CT investigation (Figure 2(a)).

On magnetic resonance imaging (MRI), GD also manifested as thin walled cystic lesions (Figure 2(c)).

Upper gastrointestinal contrast examinations performed in 3 patients documented in each case a contrast filled sack extending from the cardia region (Figures 1 and 3).

Endoscopy showed an entry at the posterior stomach wall (Figures 1 and 3).

## 4. Discussion

Our results showed that GD had a prevalence of 0.12% of all CT investigations. In addition, our series showed that GD occurred most frequently in female patients with a female : male ratio of 2.6 : 1. Furthermore, the female patients were also older. All GD were found on the posterior gastric wall.

As reported previously, the prevalence of GD varied in different investigations [1–3, 8–10]. For example, on endoscopy, GD were seen in 0.01%–0.11% of all gastroduodenal examinations [8, 9]. In autopsy, their frequency varied from 0.01 to 2.6% [1–3, 8, 9]. Upper gastrointestinal studies with oral contrast agents revealed an occurrence of stomach diverticula in 0.03%–0.1% [1, 8, 9]. In our analysis, the prevalence of GD on CT was 0.12%. Clearly, the true prevalence of GD is difficult to ascertain. There were no population-based data regarding GD in the literature. Furthermore, either the reported studies revealed clinically symptomatic diverticula or the described diverticula were detected incidentally.

Because of its rareness, CT or MRI appearances of GD have been described only sporadically [5–7]. As seen in our analysis, GD present typically as thin walled cystic lesion with air fluid level suggesting a connection to the gastrointestinal tract. However, if a diverticulum contains no air it may be misinterpreted as a different structure. For example, in one of our cases gastric diverticulum was misdiagnosed initially as an accessory spleen. As reported previously, because of its location, stomach diverticula may mimic solid or cystic lesions of the left adrenal gland [6, 7]. Other differential diagnoses include pancreatic pseudocyst or gastric wall duplication [5–7]. In these cases, three-dimensional reconstruction is helpful by identifying a connection to the stomach. Furthermore, CT investigation with oral contrast shows retention of contrast medium in the diverticula. According to the literature, on MRI, GD also manifest as fluid containing structures [7]. Upper gastrointestinal contrast examinations typically document a contrast filled sack suggesting GD [3, 4].

Interestingly, in our study, all identified diverticula were located on the posterior gastric wall in the cardia below the esophagogastral junction. According to the literature, this is the typical localization for congenital gastric diverticula [1, 4]. However, the median age of our patients was 64 years. We found no diverticula in the antrum or in the pars pylorica of the stomach.

As reported previously, clinically, most described GD were incidental findings as they were in our series [1–3, 11]. They can present with unspecific abdominal pain or dysphagia [1, 11]. However, acute complications, such as bleeding or perforation, have also been described in the literature [2, 3]. According to previous reports, GD can contain ectopic pancreatic tissue [2]. Furthermore, cases of carcinoma arising in GD have been reported previously [12].

Asymptomatic diverticula need no treatment. In cases with acute complications surgical resection of CGD is the method of choice [1].

## 5. Conclusion

The prevalence of congenital stomach diverticula encountered in our CT series is 0.12%. GD demonstrate typical CT appearances, namely, cystic lesions with thin wall and air fluid level, located in the left paravertebral region between the spleen, adrenal gland, and crus of the left diaphragm. The radiologist should be familiar with this finding to avoid possible misinterpretations.
